# Role of MMP-2 and CD147 in kidney fibrosis

**DOI:** 10.1515/biol-2022-0482

**Published:** 2022-09-14

**Authors:** Zhengyuan Cheng, Xiaojuan Zhang, Yu Zhang, Li Li, Pingsheng Chen

**Affiliations:** Department of Internal Medicine, Ma’anshan People’s Hospital Affiliated to Medical School of Southeast University, Hubei Road 45, Huashan District, Ma’anshan 243099, Anhui Province, China; Department of Nephrology, Jinling Hospital Affiliated to Nanjing University, Zhongshan East Road 305, Xuanwu District, Nanjing 210008, Jiangsu Province, China; Department of Pathology and Pathophysiology, Medical School, Southeast University, Dingjiaqiao 87, Gulou District, Nanjing 210009, Jiangsu Province, China

**Keywords:** MMP-2, CD147, hypoxia, kidney fibrosis

## Abstract

Matrix metalloproteinase-2 (MMP-2) and cluster of differentiation 147 (CD147) both play important roles in the development of kidney fibrosis, and CD147 can induce the production and activation of MMP-2. In the early stage of kidney fibrosis, MMP-2 promotes extracellular matrix (ECM) production and accelerates the development of kidney fibrosis, while in the advanced stage, MMP-2 activity decreases, leading to reduced ECM degradation and making it difficult to alleviate kidney fibrosis. The reason for the decrease in MMP-2 activity in the advanced stage is still unclear. On the one hand, it may be related to hypoxia and endocytosis, which lead to changes in the expression of MMP-2-related active regulatory molecules; on the other hand, it may be related to insufficient CD147 function. At present, the specific process by which CD147 is involved in the regulation of MMP-2 activity is not completely clear, and further in-depth studies are needed to clarify the roles of both factors in the pathophysiology of kidney fibrosis.

## 1 Introduction

Chronic kidney disease (CKD) is a worldwide public health problem threatening human health [1]. CKD is divided into five stages. In stages 1 and 2 CKD, renal function can still be compensated, but as the disease progresses to stage 5, renal function will be completely lost. Therefore, stage 5 CKD is also called end-stage renal disease (ESRD). Studies have shown that kidney fibrosis is the main pathological change in ESRD [1,2]; therefore, kidney fibrosis is considered the final manifestation of CKD [3]. Kidney fibrosis is also the result of abnormal repair after renal injury, a complex process involving renal intrinsic cells, such as endothelial cells, tubular epithelial cells, mesangial cells, and activation of the human immune system. During repair, an autoinflammatory reaction occurs, which leads to the extensive infiltration of inflammatory cells, such as macrophages and lymphocytes into renal tissue. These cells simultaneously release a large number of inflammatory mediators [4,5,6], such as tumour necrosis factors, interleukins, and chemokines (CKs), that not only aggravate kidney tissue damage but also activate transforming growth factor (TGF)-β/Smad, hypoxia-inducible factor (HIF), and other cellular signalling pathways associated with kidney fibrosis [3,7]. Ultimately, these changes lead to the substantial deposition of extracellular matrix (ECM) outside the cell and obstruction of the renal interstitial capillary bed and hypoxia, thereby promoting the development of kidney fibrosis.

As mentioned earlier, there are multiple reasons for the considerable deposition of ECM, and matrix metalloproteinase (MMP)-2, an important molecule in the MMP family, has received much attention for its role in the process of kidney fibrosis [8]. Recent studies suggest [9,10,11] that MMP-2 can promote ECM production and accelerate kidney fibrosis in the early stage; however, MMP-2 activity decreases in the advanced stage, which reduces ECM degradation and accelerates disease progression. Therefore, increasing MMP-2 activity in the advanced stage of kidney fibrosis may promote fibrosis remission. The mechanism underlying the deficient MMP-2 activity in the advanced stage of kidney fibrosis remains unknown but is absolutely related to the regulation of its activity. In addition to the classical pathway (Section 2.1), MMP-2 activity is also regulated by extracellular matrix metalloproteinase inducer (EMMPRIN), also known as cluster of differentiation 147 (CD147) [12]. CD147 is involved in a variety of pathophysiological processes [13,14], such as tumour metastasis and coronavirus disease 2019 (COVID-19) infection of the lungs. However, the mechanisms by which CD147 regulates MMP-2 activity and contributes to the development of kidney fibrosis are still undetermined. We hope this review will draw considerable attention to the study of these two molecules in kidney fibrosis.

## 2 MMP-2 and kidney fibrosis

### 2.1 MMP-2 activation

**Figure 1:**
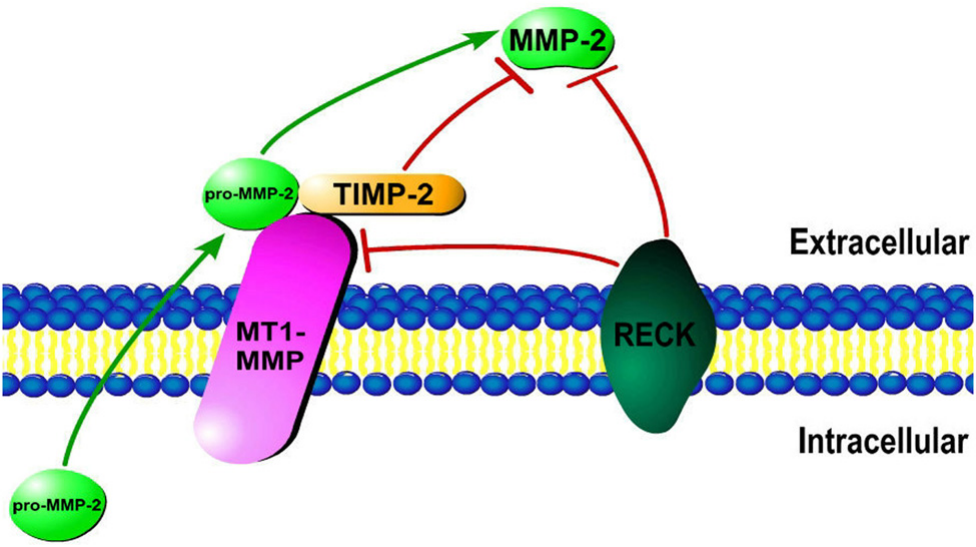
The activation of MMP-2, which occurs mainly on the cell surface and is regulated by various molecules, such as MT1-MMP, TIMP-2, and RECK.

MMP-2 is an important member of the MMP family that is released from cells in the form of pro-MMP-2, and the main regulators of MMP-2 activation include membrane type-1 MMP (MT1-MMP), a reversion-inducing cysteine-rich protein with Kazal motifs (RECK), and tissue inhibitor of matrix metalloproteinase-2 (TIMP-2). The activation of pro-MMP-2 mostly occurs on the cell surface [8], where pro-MMP-2, TIMP-2, and MT1-MMP together form a complex that enables the conversion of pro-MMP-2 to MMP-2 (Figure 1). In this process, MT1-MMP can facilitate the activation of MMP-2, and TIMP-2 has dual roles [15]: a low concentration of TIMP-2 can promote MMP-2 activation, whereas a high concentration of TIMP-2 inhibits MMP-2 activation and activity. RECK can inhibit MMP-2 activation either directly or by inhibiting MT1-MMP [16,17].

### 2.2 Role of MMP-2 in kidney fibrosis

MMP-2, which can degrade gelatine *in vitro*, is also called gelatinase A. When MMP-2 is activated, it can degrade or modify a variety of extracellular substances, including basement membrane components and a variety of ECM factors, such as collagen type I, III, IV, VI (α-chain), IX, X, XII, and V/XI and fibronectin [18], as well as the precursor pro-TGF-β1, insulin-like growth factor (IGF), fibroblast growth factor receptor 1, and other profibrotic and proinflammatory factors [19,20,21]. Fernandez-Patron and Leung reported that [22] MMP-2 can also play an anti-inflammatory role by antagonizing phospholipase A2 (PLA2); in addition, MMP-2 can promote the activation of the MMP-1, MMP-9, and MMP-13 zymogens. MMP-2 has a wide range of pathophysiological functions and is closely related to both inflammation and fibrosis. Considering that the main pathological feature of CKD is kidney fibrosis, MMP-2 is suggested to be involved in various stages of the development of kidney fibrosis.

Studies of CKD have shown that glomerular mesangial cells, renal tubular epithelial cells, and interstitial cells all produce MMP-2, which may play different roles during the initial and advanced stages of CKD. MMP-2 accelerates the development of early CKD because it can degrade the basement membrane, destroy the glomerular filtration membrane, activate TGF-β1, and promote the phenotypic transformation of tubular epithelial cells [9]; renal tubular atrophy, renal fibrosis, and excessive ECM deposition in the basement membrane and interstitium are observed in advanced CKD, suggesting that MMP-2 activity is insufficient at this stage [10]. This insufficient activity may not only lead to reduced ECM degradation and CK inactivation and to resistance to PLA2 inhibition, thus promoting the development of chronic inflammation, but also result in the insufficient degradation of endothelin-1, adrenomedullin, and calcitonin gene-related peptides by MMP-2, aggravating intrarenal arteriolar spasms and sclerosis. Tveitarås et al. found that [23] MMP-2 could alleviate kidney fibrosis in gene knockout mice. Cheng et al. and Yu et al. found that [10,24,25] in the advanced stage of renal fibrosis, exacerbated hypoxia leads to increased HIF-1α expression, active endocytosis, and changes in the cell membrane structure. Together, these events can decrease MT1-MMP expression and increase TIMP-2 and RECK expressions, thus inhibiting MMP-2 activity, making it more difficult to degrade the excess ECM deposits, and aggravating kidney fibrosis. The above facts illustrate that increasing MMP-2 activity in advanced-stage CKD may effectively alleviate kidney fibrosis. Hence, MMP-2 is a valuable target in kidney fibrosis.

## 3 Role of CD147 in kidney fibrosis

MMP-2 plays an important role in the development of kidney fibrosis, and we also briefly described the classical pathway of MMP-2 activation earlier. In fact, there is another molecule in humans, EMMPRIN (or CD147) [26], which has a significant impact on both the regulation of MMP-2 activation and the development of renal fibrosis [27,28].

### 3.1 Brief introduction to CD147

CD147 is a transmembrane (TM) glycoprotein in the immunoglobulin superfamily (Ig SF) that is widely expressed on the surface membrane of various cells [29,30]. CD147 has so far been found in many species, albeit with different names, including EMMPRIN in humans [31], OX47 in rats, and Basigin (BSG) in mice [32]. Subsequently, this protein was given the uniform name of CD147, and it is now collectively referred to as CD147, EMMPRIN, or BSG. CD147 is a single TM glycoprotein with an extracellular N-terminus and an intracellular C-terminus [33], and the gene encoding human CD147 (*BSG* gene) maps to chromosome 19p13.3 [34]. There are four BSG isomers in humans with different predominant expression patterns [35,36,37]: BSG-1 (retina), BSG-2 (heart, kidney, skeletal muscle, and testis), BSG-3, and BSG-4 (skeletal muscle, liver, lung, thyroid, and other tissues).

### 3.2 Molecular structure and glycosylation of CD147

#### 3.2.1 Molecular structure of CD147

**Figure 2:**
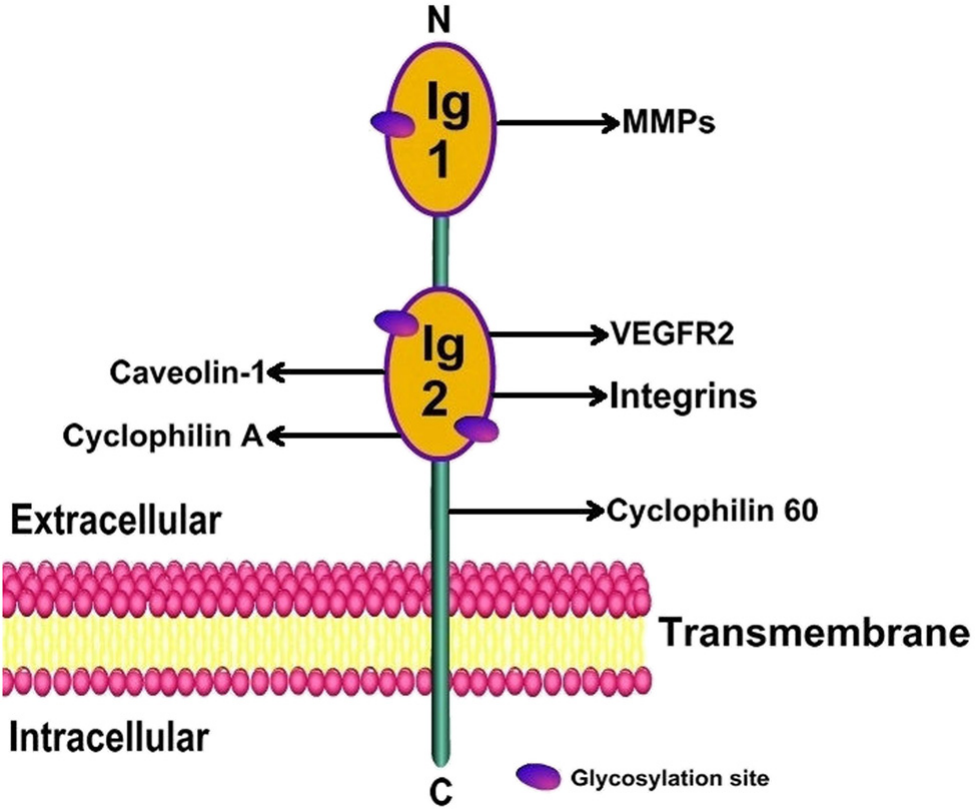
Molecular structure of BSG-2. BSG-2 contains an extracellular region, a TM region, and an intracellular region. The extracellular domain includes the Ig1 and Ig2 domains. The Ig1 domain can stimulate the production and activation of MMPs, and the Ig2 domain enables CD147 to exert different biological activities by interacting with different molecules. There are three sites in the Ig1 and Ig2 domains that can be modified by glycosylation.

Since BSG-2 is abundant in the kidney, it is used as an example within this review (Figure 2). BSG-2 mainly localizes to the cell membrane and includes three regions [33,37]: the extracellular region, TM region, and intracellular region. The extracellular region of BSG-2 includes two Ig-related domains: Ig1 and Ig2. The Ig1 domain is important for inducing the production and activation of MMPs [37], and the Ig2 domain can interact with caveolin-1, integrins (α6β1 and α3β1), vascular endothelial growth factor receptor 2 (VEGFR2), and cyclophilin A (CyPA) [37,38,39], thereby exerting biological effects. In addition, the area of the TM region near the extracellular region is the main location of CD147 binding to cyclophilin 60 (Cyp60). After CD147 binds Cyp60, this area contributes to the transport of CD147 from the Golgi body to the cell surface [39].

#### 3.2.2 Glycosylation of CD147

The extracellular segment of CD147 has three glycosylation sites [37,40,41], two in the Ig2 domain and one in the Ig1 domain (Figure 2). CD147 glycosylation allows for variance in the relative molecular mass between 32 and 65 kDa [42]. CD147 exists in both low- and high-glycosylated forms with relative molecular masses of ∼32 and 45–65 kDa, respectively. Highly glycosylated CD147 can induce cells to produce different MMPs, including MMP-1, MMP-2, and MMP-9 [42,43,44]. However, caveolin-1 can selectively bind the Ig2 domain of the low-glycosylated form of CD147, which prevents the further glycosylation needed to generate the highly glycosylated form [42]. The low-glycosylated form of CD147 is considered unable to induce the production and activation of MMPs [42,45,46].

### 3.3 Soluble CD147

CD147 is located on the cell membrane and can play a role in cells; however, it can also exist and function extracellularly in a free form called soluble CD147 (sCD147) [47,48]. There are three forms of sCD147 [49,50,51]: full-length CD147, CD147 produced by microbubble shedding, and the extracellular segment of CD147 shed from the cell surface in a process mediated by MT1-MMP. All three types of sCD147 can stimulate fibroblasts to produce MMPs and regulate the activation of MMPs, but the specific mechanism remains unclear. It is speculated that sCD147 may bind to and dimerize with CD147 on the cell membrane to enhance function. Belton et al. reported that [52] sCD147 and CD147 can bind and dimerize in uterine fibroblasts, and dimerized CD147 can stimulate an increase in MMP-1 and MMP-2 levels in uterine fibroblasts. However, whether sCD147 has the above effects on renal fibroblasts has not been investigated.

### 3.4 CD147 and kidney fibrosis

#### 3.4.1 Ig1 domain and kidney fibrosis

The process of kidney fibrosis is complex and mainly involves tissue and cell damage, the release of inflammatory mediators, inflammatory cell infiltration, epithelial–mesenchymal transition, and excessive ECM deposition, among others. During this process, CD147 can stimulate fibroblasts to produce MMP-2 and MMP-9 [42,46], and this stimulatory effect is mainly related to the Ig1 domain of CD147. These MMPs can activate the TGF-β/Smad, Notch, and other signalling pathways [53,54], which can promote the development of renal fibrosis. In addition, Kato et al. found that [55] during renal fibrosis, CD147 on fibroblasts cooperates with MMPs to induce hyaluronan production on tubule epithelial cells, which can promote the conversion of fibroblasts into myofibroblasts. However, studies in mouse models revealed milder renal fibrosis in BSG−/− mice than in wild-type mice because of reduced MMP production [55]. In addition, the renal tubular epithelial cells in BSG−/− mice had a weaker response to TGF-β than those in wild-type mice. It is unclear why the Ig1 domain of CD147 activates MMPs, but this activation has been suggested to relate to the interaction between Aspartate^179^ (Asp^179^) in the Ig1 domain and the metal ion-dependent adhesion site in the βA domain of the integrin β1 subunit [56,57].

#### 3.4.2 Ig2 domain and kidney fibrosis

The Ig2 domain is the most functionally complex domain in CD147; as mentioned earlier, this domain can bind to caveolin-1, integrins, VEGFR2, CyPA, and others to exert biological effects. The effects of this domain on kidney fibrosis after binding caveolin-1 will be considered in the next section.

At present, the Ig2 domain of CD147 is known to interact with α3β1 and α6β1 integrins to function within and between cells by forming complexes [56,57,58]. Integrins often advance the development of renal fibrosis [59,60] by accelerating fibroblast activation in renal tissue [61]. Furthermore, in the TGF-β/Smad signalling pathway, TGF-β can stimulate normal glomerular integrin synthesis and promote cell adhesion to matrix proteins mediated by integrin, thereby facilitating ECM deposition in the glomerulus [62,63]. All these factors contribute to the aggravation of renal fibrosis.

Recent studies have shown that both CD147 and sCD147 can promote VEGF production, and the binding of VEGF to VEGFR2 can promote angiogenesis and alleviate kidney fibrosis [64]. However, in the process of kidney fibrosis, the VEGF/VEGFR-2 signalling pathway can be inhibited by the active endocytosis and degradation of VEGFR-2, thus decreasing angiogenesis and accelerating renal fibrosis [65,66].

CyPA, an intracellular protein ubiquitously expressed in various mammalian cell types, is the main intracellular receptor of the immunosuppressant cyclosporine A (CsA). CyPA can be secreted from cells upon stimulation by infection, hypoxia, and oxidative stress [67,68,69]. As the main signal transduction receptor of extracellular CyPA, CD147 mediates the chemotactic activity of cyclophilin on various immune cells and promotes and amplifies the inflammatory response [70,71]. The signalling pathways related to CyPA/CD147 include the protein kinase B, ERK1/2, and nuclear factor κB (NF-κB) pathways, among others [72,73]. The inhibition of CyPA has been suggested to attenuate myocardial fibrosis [74], liver fibrosis [75], and pulmonary arterial hypertension [76], indicating that CyPA can promote the occurrence of these diseases. Interestingly, the study by Leong et al. suggested that [77] CyPA can promote an inflammatory response and acute renal injury during renal ischaemia‒reperfusion but not in kidney fibrosis. Therefore, further investigation is needed to fully elucidate the role of CyPA in kidney fibrosis.

The earlier discussion highlights that different domains of CD147 can interact with different molecules, so the mechanism by which CD147 affects renal fibrosis is complex and results from the actions of all three CD147 domains. These concepts also suggest that CD147 may exert specific functions under the control of complex and elaborate regulatory mechanisms, but further study is required.

## 4 Pathway by which MMP-2 activity is regulated by CD147

Another important question relates to how CD147 regulates MMP-2 activity. Although the answer to this question is still unclear, some data suggest that it is possible that CD147 interacts with other molecules to regulate MMP-2 activity rather than controlling it directly. Santiago-Gómez et al. found that [78] β1 integrin can form a complex with CD98 and CD147, thereby inactivating CD147 and leading to decreased MMP-2 activity. In addition, Grass et al. found that [79] hyaluronan-CD44 can lead to the oligomerization of CD147-containing complexes, which in turn regulates MT1-MMP activity, an important molecule for MMP-2 activation. CD147 has also been shown to regulate the cellular synthesis of MT1-MMP [80,81]. Therefore, CD147 potentially regulates MMP-2 activity through its interactions with MT1-MMP.

**Figure 3:**
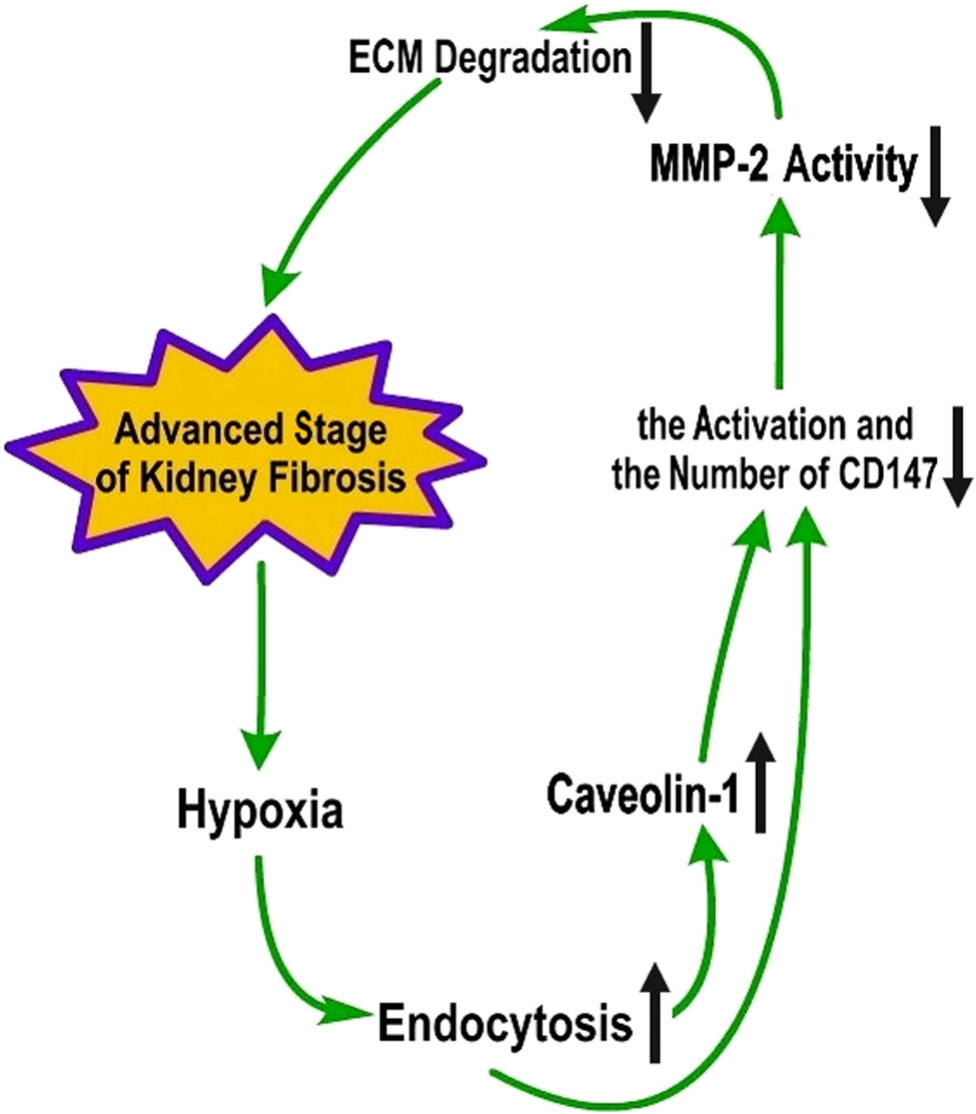
Possible pathway by which CD147 regulates MMP-2 activity in the advanced stage of kidney fibrosis. Obvious hypoxia in the advanced stage of kidney fibrosis leads to active endocytosis and an increase in caveolin-1 expression, thus decreasing the activity and amount of CD147, further attenuating MMP-2 activity, reducing ECM degradation, and exacerbating kidney fibrosis.

How does CD147 regulate MMP-2 activity during kidney fibrosis? There are currently few studies that help answer this question. As mentioned earlier [9,10,11], MMP-2 activity is increased in the early stage of kidney fibrosis, which can promote ECM production and accelerate kidney fibrosis. Whereas in the advanced stage, MMP-2 activity decreases, reducing ECM degradation. CD147 may also be involved in the changes in MMP-2 activity at different stages of kidney fibrosis. In the early stage of kidney fibrosis, CD147 can promote disease progression by enhancing the production and activation of MMP-2 [42,46]. CD147 is thought to be defective in the advanced stage of kidney disease, resulting in insufficient MMP-2 activity and reduced ECM degradation, making it difficult to alleviate kidney fibrosis. There have been few studies on the specific pathway by which CD147 regulates MMP-2 activity in renal fibrosis; nonetheless, by combining the results of earlier studies by our group and others, we propose one possible regulatory pathway in the advanced stage of kidney fibrosis. As mentioned earlier, high glycosylation of CD147 promotes MMP production and activation, while caveolin-1 plays an important regulatory role in the transition from low to high glycosylation [42]. Moreover, hypoxia is considered an important factor that contributes to the development and aggravation of kidney fibrosis [82,83]; therefore, Fine et al. proposed the famous “chronic hypoxia theory” [84]. Our group also found [10,22,23] significant hypoxia in a rat model of advanced renal fibrosis; moreover, we found that caveolin-1 expression was upregulated and that endocytosis was active in both this model and hypoxic cultured renal tubular epithelial cells. Based on the currently available data, the following model is proposed. The upregulation of caveolin-1 expression affects the hyperglycosylation of CD147, leading to insufficient MMP-2 activation; in addition, the hypoxic conditions in renal fibrosis evoke intense endocytic activity [10,25], which drastically alters the structure of the cell membrane [85,86]. As CD147 localizes to the cell membrane, these structural changes can lead to the internalization of CD147 by endocytic vesicles, resulting in a decreased amount of CD147 at the surface. Accordingly, the change in membrane structure can also affect CD147 function, thereby negatively impacting MMP-2 activation and thus reducing ECM degradation in the advanced stage of renal fibrosis (Figure 3). However, additional experimental evidence is required to validate the above pathway, which represents only a possibility based on existing data.

## 5 Conclusions

**Figure 4:**
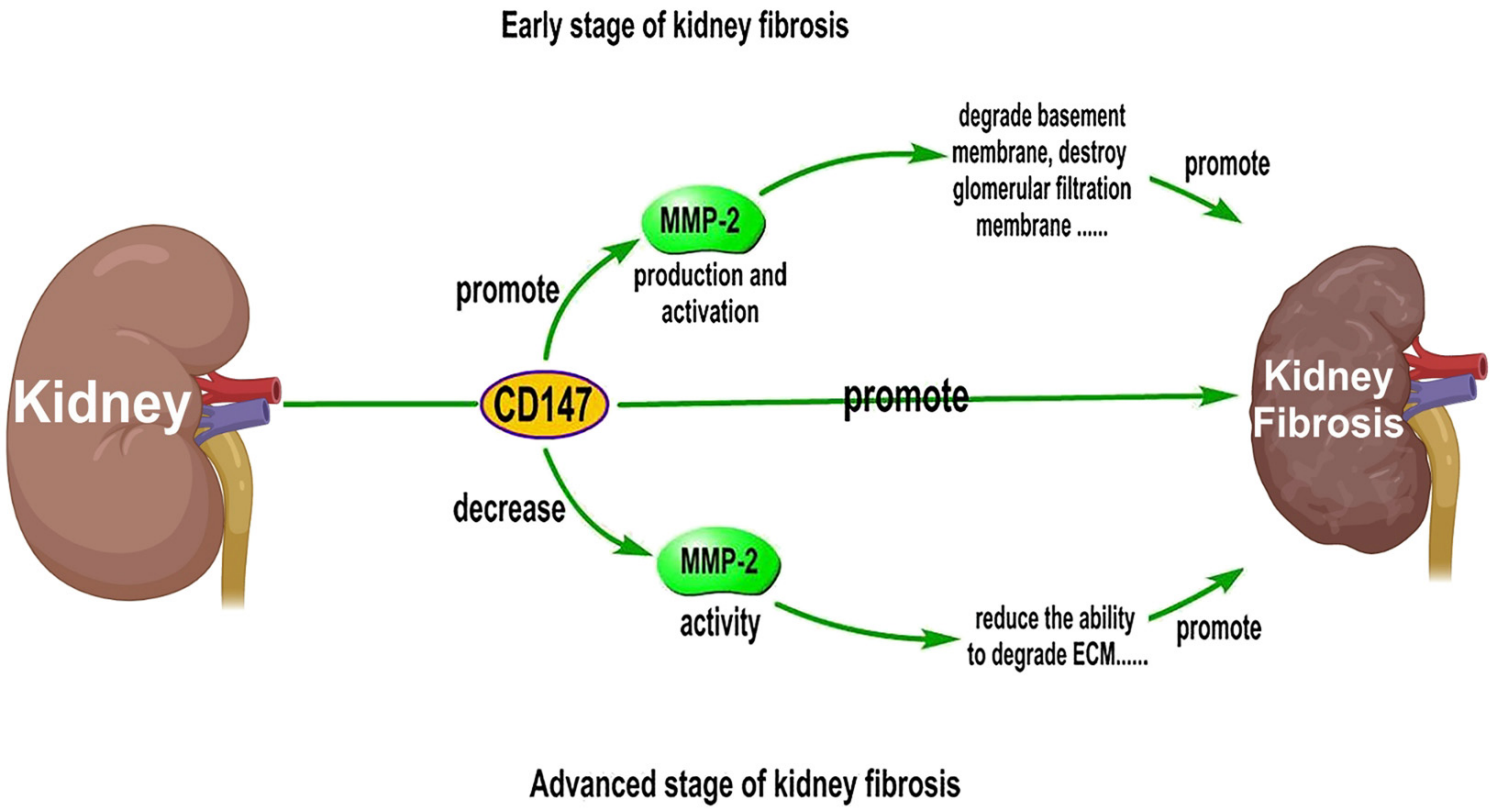
Differential effects of CD147 on MMP-2 activity at different stages of kidney fibrosis. MMP-2 activity can be increased in the early stage of kidney fibrosis and decreased in the advanced stage of kidney fibrosis. These changes accelerate kidney fibrosis and make it difficult to alleviate the disease.

This review focuses on the roles of MMP-2 and CD147 in kidney fibrosis, and current data suggest that these factors play complex roles. CD147 can not only directly promote the development of kidney fibrosis but also differentially regulate MMP-2 activity at different stages of renal fibrosis (Figure 4). However, the specific regulatory mechanism is unclear. If the regulatory mechanism can be clarified, CD147 could be targeted in the future to regulate MMP-2 activity as a new strategy for the treatment of kidney fibrosis.
